# Does insulin resistance influence neurodegeneration in non-diabetic Alzheimer’s subjects?

**DOI:** 10.1186/s13195-021-00784-w

**Published:** 2021-02-17

**Authors:** Grazia Daniela Femminella, Nicholas R. Livingston, Sanara Raza, Thalia van der Doef, Eleni Frangou, Sharon Love, Gail Busza, Valeria Calsolaro, Stefan Carver, Clive Holmes, Craig W. Ritchie, Robert M. Lawrence, Brady McFarlane, George Tadros, Basil H. Ridha, Carol Bannister, Zuzana Walker, Hilary Archer, Elizabeth Coulthard, Ben Underwood, Aparna Prasanna, Paul Koranteng, Salman Karim, Kehinde Junaid, Bernadette McGuinness, Anthony Peter Passmore, Ramin Nilforooshan, Ajayverma Macharouthu, Andrew Donaldson, Simon Thacker, Gregor Russell, Naghma Malik, Vandana Mate, Lucy Knight, Sajeev Kshemendran, Tricia Tan, Christian Holscher, John Harrison, David J. Brooks, Clive Ballard, Paul Edison

**Affiliations:** 1 Division of Neurology, Neurology Imaging Unit, Department of Brain Sciences, Imperial College London, 1st Floor B Block, Hammersmith Hospital Campus, Du Cane Road, London, W12 0NN UK; 2 University of Oxford, Oxford, UK; 3 University of Southampton, Southampton, UK; 4 University of Edinburgh, Edinburgh, UK; 5 South West London and St George’s Mental Health NHS Trust, London, UK; 6 Southern Health NHS Foundation Trust, Southampton, UK; 7 Heart of England NHS Foundation Trust, Birmingham, UK; 8 Brighton and Sussex University Hospital Trust, Brighton, UK; 9 King’s College London, London, UK; 10 Mental Health Unit, St. Margaret’s Hospital, Epping, Essex, UK; 11 North Bristol NHS Trust, Bristol, UK; 12 Cambridgeshire and Peterborough NHS Foundation Trust, Cambridge, UK; 13 Black Country Partnership NHS Foundation Trust, Wolverhampton, UK; 14 Northamptonshire Healthcare NHS Foundation Trust, Northampton, UK; 15 Lancashire Care NHS Foundation Trust, Preston, UK; 16 Nottinghamshire Healthcare NHS Foundation Trust, Nottingham, UK; 17 Queen’s University Belfast, Belfast, UK; 18 Surrey and Borders Partnership NHS Foundation Trust, Chertsey, UK; 19 NHS Ayrshire and Arran, Kilmarnock, UK; 20 NHS Lanarkshire, Airdrie, UK; 21 Derbyshire Healthcare NHS Foundation Trust, Derby, UK; 22 Bradford District Care NHS Foundation Trust, Bradford, UK; 23 North West Boroughs Partnership NHS Foundation Trust, Warrington, UK; 24 Cornwall Partnership NHS Foundation Trust, Redruth, UK; 25 Somerset Partnership NHS Foundation Trust, South Petherton, UK; 26 South Staffordshire and Shropshire Healthcare NHS Foundation Trust, Shrewsbury, UK; 27 Research and Experimental Center, Henan University of Chinese Medicine, Zhengzhou, China; 28 Alzheimer Center Amsterdam, Department of Neurology, Amsterdam Neuroscience, Amsterdam UMC, Vrije Universiteit Amsterdam, Amsterdam, Netherlands; 29 Newcastle University, Newcastle upon Tyne, UK; 30 University of Exeter Medical School, Exeter, UK

**Keywords:** Alzheimer’s disease, Insulin resistance, Magnetic resonance imaging, Positron emission tomography imaging

## Abstract

**Background:**

Type 2 diabetes is a risk factor for Alzheimer’s disease (AD), and AD brain shows impaired insulin signalling. The role of peripheral insulin resistance on AD aetiopathogenesis in non-diabetic patients is still debated. Here we evaluated the influence of insulin resistance on brain glucose metabolism, grey matter volume and white matter lesions (WMLs) in non-diabetic AD subjects.

**Methods:**

In total, 130 non-diabetic AD subjects underwent MRI and [18F]FDG PET scans with arterial cannula insertion for radioactivity measurement. T1 Volumetric and FLAIR sequences were acquired on a 3-T MRI scanner. These subjects also had measurement of glucose and insulin levels after a 4-h fast on the same day of the scan. Insulin resistance was calculated by the updated homeostatic model assessment (HOMA2). For [18F]FDG analysis, cerebral glucose metabolic rate (rCMRGlc) parametric images were generated using spectral analysis with arterial plasma input function.

**Results:**

In this non-diabetic AD population, HOMA2 was negatively associated with hippocampal rCMRGlc, along with total grey matter volumes. No significant correlation was observed between HOMA2, hippocampal volume and WMLs.

**Conclusions:**

In non-diabetic AD, peripheral insulin resistance is independently associated with reduced hippocampal glucose metabolism and with lower grey matter volume, suggesting that peripheral insulin resistance might influence AD pathology by its action on cerebral glucose metabolism and on neurodegeneration.

**Supplementary Information:**

The online version contains supplementary material available at 10.1186/s13195-021-00784-w.

## Background

Alzheimer’s disease (AD) is the most common type of dementia, affecting nearly 40 million people worldwide, and is a major public health emergency of this century. Neuropathologically, AD is characterised by extracellular accumulation of amyloid-beta (Aβ) peptides, also known as plaques, and intracellular neurofibrillary tangles (NFT), aggregates resulting from tau protein hyperphosphorylation [1].

The accumulation of these neurotoxic peptides in the cerebral cortex ultimately leads to neuronal death and gradual and progressive decline in cognitive function. Several interventional trials have targeted these two major pathological mechanisms in AD, but unfortunately, these therapeutic strategies have not resulted in successful treatment [2], although some studies are still ongoing [3]. On the other hand, the most successful preventive strategies in AD are linked to targeting modifiable risk factors [4, 5]. Among them, growing evidence indicates a close relationship between type 2 diabetes mellitus and AD. Type 2 diabetes is a risk factor for AD and epidemiological data show an almost doubled risk for AD in diabetic patients, compared with non-diabetic subjects [6].

Although the exact underlying mechanism is still unknown, numerous studies have suggested that insulin resistance is a key risk factor for AD [7, 8]. Insulin resistance is defined as reduced tissue responsiveness to the action of insulin. In AD, peripheral insulin resistance is more common compared to control subjects [9]; in the Rotterdam study, insulin resistance has been associated with an increased risk of AD at 3 years, even in the absence of type 2 diabetes [6].

In addition to its peripheral actions, insulin plays an important role in brain function. Neuronal insulin receptor activation can induce dendritic sprouting, neuronal stem cell activation, cell growth and repair [10, 11]. Insulin signalling induces the expression of the insulin-degrading enzyme (IDE), which is involved in both insulin and Aβ degradation; hyperinsulinemia might determine a competitive inhibition for IDE-dependent Aβ degradation, leading to Aβ accumulation [12].

Insulin also seems to have neuroprotective effects by regulating phosphorylated tau levels. Insulin resistance is associated with elevated levels of proinflammatory cytokines such as C-reactive protein and IL-6, which are linked to Aβ deposition in the brain [13]. Moreover, insulin improves brain functions such as attention, memory and cognition in humans [14–16]; indeed, the insulin receptor is abundant in the hippocampus along with cortex and thalamus [17], and in critical areas for metabolic control such as the hypothalamus. Recent evidence from a trial of intranasal insulin in AD subjects, however, did not show benefit in terms of cognitive outcomes at twelve months [18].

It has been shown that systemic insulin resistance and hyperinsulinemia might reduce brain insulin levels through a compensatory mechanism involving insulin receptors’ reduction in the blood-brain barrier [19]. Furthermore, insulin levels in cerebrospinal fluid (CSF) are correlated with those in plasma, suggesting that most insulin in the brain derives from circulating insulin [20]. Therefore, evidence suggests that peripheral insulin resistance is associated with impaired brain insulin action, although the mechanisms remain to be elucidated. Recent evidence suggests that brain insulin resistance could be an important trigger in the development of AD neuropathology [8, 21]. Interestingly, recent study has shown that there is significant improvement in cognition in patients receiving anti-diabetic agent dulaglutide [22] in diabetic patients suggesting the potential role of insulin resistance in cognitive impairment in diabetic patients. However, the role of peripheral insulin resistance in non-diabetic AD subjects has not been explored.

In this study, we sought to evaluate the possible association of peripheral insulin resistance with markers of synaptic function and neurodegeneration, i.e. cerebral glucose metabolic rate and MRI volume changes, as well as cerebral small vessel disease, i.e. white matter lesion (WML) volume, in non-diabetic Alzheimer’s disease subjects.

## Methods

### Study population

In total, 130 AD subjects were enrolled as a part of the Evaluating Liraglutide in Alzheimer’s Disease (ELAD) study [23]. Inclusion and exclusion criteria are detailed in Supplemental Table 1. All subjects had a diagnosis of probable AD according to National Institute on Ageing–Alzheimer’s Association (NIA-AA) criteria or National Institute of Neurological and Communicative Disorders and Stroke–Alzheimer’s Disease and Related Disorders Association (NINCDS-ADRDA) criteria. Patients who had diabetes mellitus were excluded.

All subjects had clinical and neuropsychological assessment, including the Mini-Mental State Examination (MMSE), Alzheimer’s Disease Assessment Scale-Cognitive Subscale (ADAS-Cog), Clinical Dementia Rating Sum of Boxes (CDR-SoB), Alzheimer’s Disease Cooperative Study-Activities Of Daily Living (ADCS-ADL), Geriatric Depression Scale (GDS), Controlled Oral Word Association Test (COWAT), Category Fluency Test (animals) and the Neuropsychiatric Inventory (NPI). Furthermore, enrolled subjects had a brain magnetic resonance imaging (MRI) scan and a brain [18F]-fluorodeoxyglucose (FDG) positron emission tomography (PET) with arterial input analysis. Glucose and insulin levels were measured on plasma after a 4-h fast on the day of the visit for the brain MRI and [18F]FDG PET scan with arterial cannulation. The homeostatic model assessment (HOMA) was performed to determine insulin resistance; we used HOMA2, the updated HOMA model [24]. This model can be used to determine insulin sensitivity (%S) and β-cell function (%β) from paired fasting plasma glucose and specific insulin, or C-peptide. In our study, we used insulin levels. Body mass index was calculated as weight in kilogrammes/height in metres squared.

### MRI scans

MRI scans were acquired on a Verio, 3-T clinical MRI system (Siemens, VB19) using a 32-channel head coil and included a T1-weighted magnetization-prepared rapid-acquisition gradient echo sequence (repetition time = 2400 milliseconds [ms], echo time = 3.06 ms, flip angle of 9, inversion time = 900 ms, matrix = 256 × 256) generating 1-mm^3^ isotropic voxels, for co-registration with the PET images for regional PET analysis. T2-weighted MRI sequences were also acquired to evaluate vascular and other structural abnormalities.

### [18F]FDG PET scans

All subjects were scanned using a Siemens ECAT EXACT HR+ scanner. Subjects were required to fast for 4 h before a bolus injection of 185 ± 8 MBq of [18F]FDG. A 60-min dynamic emission scan was acquired using a predefined protocol. In all subjects, a radial artery was cannulated. Continuous online sampling was performed for 15 min and then discrete blood samples were taken at baseline and at 5, 10, 15, 20, 30, 40, 50 and 60 min for radioactivity measurement. Haematocrit was estimated from the baseline blood sample and plasma glucose levels were measured in selected samples.

### Analysis of [18F]FDG PET data

Absolute regional cerebral metabolic rate of glucose consumption (rCMRGlc) parametric maps were generated with spectral analysis using an arterial input function and a lumped constant of 0.48. Parametric maps of cerebral glucose metabolism were co-registered to the individuals’ MRIs and spatially transformed into MNI space using SPM8. Object maps were created by segmenting MRIs into grey matter, white matter and CSF. This binarised grey matter map was convolved with the probabilistic brain atlas to create individualised object maps of volumes of interest, as previously described [25]. We then sampled frontal, temporal, parietal and occipital cortical regions. To further evaluate the influence of insulin resistance on subcortical regions and medial temporal lobe structures, anterior cingulate, posterior cingulate cortex, thalamus, striatum, hippocampus and medial temporal lobe structures were sampled.

### Analysis of hippocampal volume, grey matter volumes and white matter lesion volumes

The hippocampal volume was calculated using FreeSurfer (Harvard Medical School; surfer.nmr.mgh.harvard.edu) on T1-weighted images. Total (cortical and subcortical) grey matter volumes were also calculated using FreeSurfer on T1-weighted scans. Hyperintensity volumetrics, determined as white matter lesions (WMLs), were segmented by the lesion growth algorithm [26] as implemented in the LST toolbox version 3.0.0 (www.statisticalmodelling.de/lst.html) for SPM. This algorithm segments T2 hyperintense lesions using T1 and FLAIR images based on a threshold (*κ*) for transformed intensities set by the operator. After visual inspection of a subset of 10 lesion maps at different *κ*, the threshold was established at 0.3.

### Statistical analysis

All statistical analyses were performed using Statistical Package for the Social Sciences SPSS version 24 (SPSS Inc., Chicago, IL, USA). The Shapiro–Wilk test was employed to assess normality of the variables. HOMA2 was log transformed to approximate a normal distribution. For descriptive statistics, categorical variables were presented as number of cases or percentage. 25th and 75th percentile values of HOMA2 were calculated and groups falling in the lower (Q1) and upper (Q4) quartiles were compared, where Q1 includes HOMA2 < 25th percentile and Q4 includes HOMA2 > 75th percentile. For the comparison of two groups, two-tailed independent Student’s *t* test was employed for the continuous normally distributed variables. Otherwise, the Mann-Whitney *U* test was used. Pearson correlation coefficients was used to test the linear association between HOMA2, cognitive measures, rCMRGlc, hippocampal, total grey matter and WML volume. Significant associations between HOMA2 and imaging measures were tested for quantitative relationship by multiple regression analysis. Significance was set at a *p* value < 0.05.

## Results

The demographic characteristics of the population are shown in Table 1. The mean age of our AD population was 71.8 ± 7 years (age range 55–85 years). The mean Mini-Mental State Examination scores (MMSE) were 24.1 ± 3.1, mean ADAS-Cog was 31.9 ± 9.9, with a mean Clinical Dementia Rating scale (sum of boxes) of 3.6 ± 1.8. On average, scores on the 30-item Geriatric Depression Scale were low suggesting that these subjects were not depressed, and one quarter of the study population was on antidepressants. Phonetic and semantic scores were below normative thresholds [27]. The great majority of subjects (82.3%) were on treatment with acetylcholine esterase inhibitors.

**Table 1:** Baseline characteristics of subjects

Age, years	71.8 ± 7.0
Gender, M/F	80/50
BMI, kg/m^2^	25.9 ± 4.3
Plasma glucose, mmol/L	4.9 ± 0.5
Plasma insulin, mIU/L	8.4 ± 7.3
HbA1c, mmol/mol	37.1 ± 6.6
HOMA2, mass units	1.1 ± 0.9
HOMA2%β	98.6 ± 63.5
HOMA2%S	147.6 ± 105.1
Total serum cholesterol, mmol/L	4.9 ± 1.3
LDL cholesterol, mmol/L	2.9 ± 1.0
HDL cholesterol, mmol/L	1.7 ± 0.6
Serum triglycerides, mmol/L	1.6 ± 0.8
On treatment with AchE-I (% of total)	82.9
On treatment with antidepressants (% of total)	25.5
MMSE	24.1 ± 3.1
CDR-SoB	3.6 ± 1.8
ADAS-Cog	31.9 ± 9.9
GDS	5.5 ± 4.7
ADCS—ADL	66.6 ± 9.1
COWAT average (total acceptable words)	10.2 ± 4.8 (30.1 ± 13.6)
Category fluency test (animals)	10.6 ± 5.1
NPI	9.6 ± 11.6
NPI—caregiver distress	5.0 ± 6.1
Anterior cingulate rCMRGlc, μmol/g/min	0.27 ± 0.05
Posterior cingulate rCMRGlc, μmol/g/min	0.30 ± 0.05
Frontal lobe rCMRGlc, μmol/g/min	0.31 ± 0.05
Temporal lobe rCMRGlc, μmol/g/min	0.23 ± 0.04
Parietal lobe rCMRGlc, μmol/g/min	0.26 ± 0.05
Occipital lobe rCMRGlc, μmol/g/min	0.28 ± 0.05
Medial Temporal lobe rCMRGlc, μmol/g/min	0.19 ± 0.03
Hippocampal rCMRGlc, μmol/g/min	0.19 ± 0.03
Thalamus rCMRGlc, μmol/g/min	0.25 ± 0.05
Striatum rCMRGlc, μmol/g/min	0.30 ± 0.05
Hippocampal volume, mm^3^	3062.2 ± 458.6
White matter lesions volume, ml	6.79 ± 7.7
Total grey matter volume, mm^3^	556,401.9 ± 52,622.8

Continuous data are shown as mean ± standard deviation

These subjects had normal fasting mean levels of plasma glucose, insulin and HbA1c, indicating a good euglycemic control in this non-diabetic population. The average BMI was 25.9 ± 4.3. The mean HOMA2 index in our group was 1.1 ± 0.9, with HOMA %β (steady-state β cell function as percentages of a normal reference population) being 98.6 ± 63.5 and HOMA %S (and insulin sensitivity as percentages of a normal reference population) was 147.6 ± 105.1.

rCMRGlc in the different ROIs, hippocampal volumes, WML and grey matter volumes are detailed in Table 2. In particular, mean hippocampal rCMRGlc was 0.19 ± 0.03 μmol/g/min, total grey matter volume was 556,401.9 ± 52,622.8 mm^3^, total (left and right) hippocampal volume was 3062.2 ± 458.6 mm^3^ and WML volume was 6.79 ± 7.7 ml.

**Table 2:** Comparison between Q1 and Q4 AD subjects

	Q1 ***N*** = 32	Q4 ***N*** = 32
Age, years	72.3 ± 5.6	71.5 ± 7.6
Gender, M/F	19/13	20/12
BMI, kg/m^2^	23.6 ± 3.7	27.4 ± 4.8 *
Plasma glucose, mmol/L	4.6 ± 0.5	5.2 ± 0.6 *
Plasma insulin, mIU/L	2.9 ± 0.8	17.5 ± 9.7 *
HbA1c, mmol/mol	36.9 ± 4.0	38.0 ± 5.4
HOMA2, mass units	0.37 ± 0.1	2.2 ± 1.1 *
HOMA2%β	58.5 ± 18.2	160.3 ± 99.4 *
HOMA2%S	295.9 ± 100.6	52.6 ± 16.6 *
Total serum cholesterol, mmol/L	5.1 ± 1.4	4.4 ± 0.9
LDL cholesterol, mmol/L	3.0 ± 1.0	2.3 ± 0.8 *
HDL cholesterol, mmol/L	1.9 ± 0.6	1.6 ± 0.7 *
Serum triglycerides, mmol/L	1.2 ± 0.5	1.7 ± 0.6 *
On statin treatment, %	40.6	53.1
On antihypertensive treatment, %	28.1	37.5
MMSE	23.8 ± 3.0	23.6 ± 2.7
CDR-SoB	3.6 ± 1.4	3.5 ± 1.5
ADAS-Cog	33.8 ± 12.0	32.5 ± 9.1
GDS	5.7 ± 4.2	5.4 ± 4.9
ADCS—ADL	68.7 ± 7.3	66.6 ± 8.8
COWAT average (total acceptable words)	10.8 ± 5.5 (29.8 ± 14.2)	9.6 ± 4.2 (29 ± 13.3)
Category fluency test (animals)	10.5 ± 5.0	10.8 ± 5.4
NPI	8.1 ± 9.1	11.2 ± 15.0
NPI—caregiver distress	4.3 ± 4.4	5.9 ± 9.1
On treatment with AchE-I, %	77.4	90.6
On treatment with antidepressants, %	25	21.9
Anterior cingulate rCMRGlc, μmol/g/min	0.28 ± 0.03	0.27 ± 0.04
Posterior cingulate rCMRGlc, μmol/g/min	0.30 ± 0.04	0.30 ± 0.05
Frontal lobe rCMRGlc, μmol/g/min	0.31 ± 0.03	0.30 ± 0.05
Temporal lobe rCMRGlc, μmol/g/min	0.23 ± 0.03	0.23 ± 0.03
Parietal lobe rCMRGlc, μmol/g/min	0.26 ± 0.04	0.26 ± 0.04
Occipital lobe rCMRGlc, μmol/g/min	0.28 ± 0.04	0.27 ± 0.04
Medial temporal lobe rCMRGlc, μmol/g/min	0.20 ± 0.02	0.19 ± 0.03 *
Hippocampal rCMRGlc, μmol/g/min	0.21 ± 0.02	0.19 ± 0.03 *
Thalamus rCMRGlc, μmol/g/min	0.25 ± 0.03	0.24 ± 0.05
Striatum rCMRGlc, μmol/g/min	0.31 ± 0.03	0.30 ± 0.06
Hippocampal volume, mm^3^	3183.0 ± 437.9	3038.9 ± 392.4
White matter lesions volume, ml	4.6 ± 4.6	6.1 ± 6.2
Total grey matter volume, mm^3^	563,626.3 ± 44,128.6	539,331.9 ± 44,547.8 *

* *p* < .05 for Q1 vs Q4 on independent sample *t*-test or Mann-Whitney *U* test

rCMRGlc showed significant inverse correlation with scores on the ADAS-Cog examination in the cortical ROIs in the frontal (Pearson’s *r* = −0.36 [− 0.52–0.18] *p* < 0.00), temporal (Pearson’s *r* = − 0.45 [− 0.62–0.30] *p* < 0.00), parietal (Pearson’s *r* = − 0.48 [− 0.62–0.34] *p* < 0.00) and occipital lobes (Pearson’s *r* = − 0.33 [− 0.49–0.15] *p* = 0.01). ADAS-Cog was also negatively correlated with hippocampal volume (Pearson’s *r* = − 0.30 [− 0.48–0.10] *p* < 0.00).

**Fig. 1:**
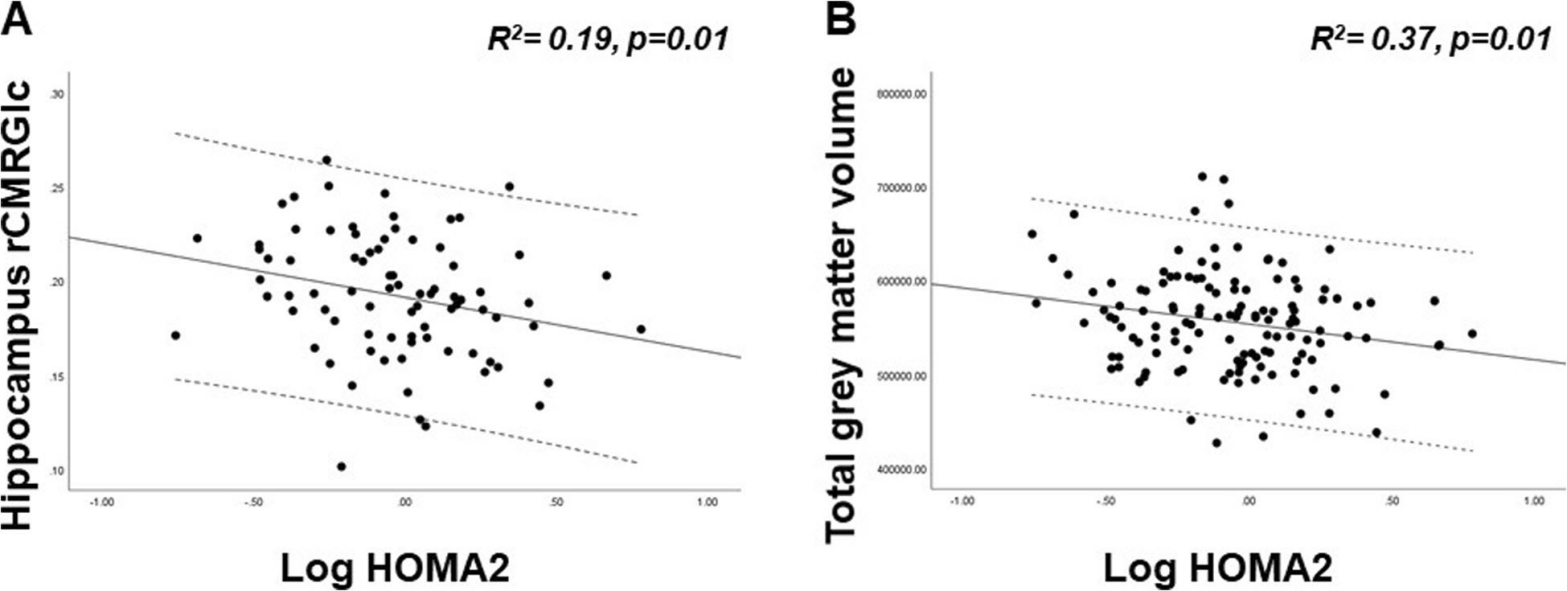
Multiple linear regression between HOMA2, hippocampal rCMRGlc and total grey matter volume. **a** The inverse association between log transformed HOMA2 hippocampal rCMRGlc, in a multiple regression model corrected by age and gender (*R*^2^ = 0.19, *p* = 0.01). Log transformed HOMA2 was negatively associated with total grey matter volumes (*R*^2^ = 0.37, *p* = 0.01), in a multiple regression model corrected by age and gender (**b**). rCMRGlc is expressed in μmol/g/min, total grey matter volumes are expressed in mm^3^

Linear correlation between log transformed HOMA2, rCMRGlc in the predefined cortical regions, hippocampal, total grey matter and WMLs was tested with Pearson correlation coefficient. HOMA2 showed a significant inverse correlation with rCMRGlc in the hippocampus (Pearson’s *r* = − 0.26 [− 0.45–0.07] *p* < 0.02) and total grey matter volume (Pearson’s *r* = − 0.23 [− 0.42–0.04] *p* < 0.04). No significant correlation was observed between HOMA2 and hippocampal volume. Bias corrected and accelerated bootstrap 95% CIs are reported in square brackets. On multiple regression analysis models corrected by age and gender, log transformed HOMA2 was independently associated with hippocampal rCMRGlc (*R*^2^ = 0.19, *p* = 0.01) and with total grey matter volume (*R*^2^ = 0.37, *p* = 0.01) (Fig. 1).

To test for an effect of disease severity on the association between HOMA2, hippocampal rCMRGlc and total grey matter volume, we further corrected our models by CDR scores: in these models, CDR scores were not significant predictors of hippocampal rCMRGlc (*R*^2^ = 0.24, *p* = 0.01 for the model) or of total grey matter volume (*R*^2^ = 0.38, *p* = 0.01 for the model).

To take into account of the atrophy, when we created the individualised object map, we multiplied the atlas with individual subject’s grey matter. This ensures that we are not sampling any regions outside the grey matter. Moreover, in order to take into account the effects of atrophy on FDG measures, we built a multiple regression model to test the association between HOMA2 and hippocampal rCMRGlc corrected by age and total grey matter volume. In this model, total grey matter volume was not a significant predictor of hippocampal rCMRGlc (*R*^2^ = 0.17, *p* = 0.02). When hippocampal rCMRGlc was normalised by individual hippocampal volumes, HOMA2 remained a significant predictor, even correcting by age (*R*^2^ = 0.15, *p* = 0.02).

Interestingly, WML volume was not correlated with HOMA2, but it positively correlated with serum triglyceride levels. In a multiple regression model, serum triglyceride levels were significant predictors of WMLs also after correcting by age and gender (*R*^2^ = 0.22, *p* = 0.00) (Supplemental Fig. 1).

To compare the subjects with a higher HOMA2 index versus those with a lower index, we divided the population into quartiles of HOMA2 index distribution and compared the group in the first quartile (Q1, or below 25th percentile) with the group in the fourth quartile (Q4, or above 75th percentile). The Q1 group was composed of the subjects (*n* = 32) whose HOMA2 was less than 0.54, while the Q4 group (*n* = 32) comprised participants whose HOMA2 was above 1.39.

We then compared the biomarkers and cognitive measures in these two groups (Table 2). Q4 subjects showed significantly reduced rCMRGlc in the hippocampus (*p* = 0.04) and in the MTL (*p* = 0.04) compared with Q1 subjects. Moreover, total grey matter volume was significantly reduced in Q4 subjects compared with Q1 (*p* = 0.03 and *p* = 0.01, respectively). Hippocampal volume and the volume of WMLs were not significantly different between the two groups. Fasting plasma glucose levels were 4.6 ± 0.5 mmol/L and 5.2 ± 0.6 mmol/L in the Q1 and Q4 groups, respectively (*p* < 0.05) and fasting plasma insulin levels were 2.9 ± 0.8 mIU/Lin Q1 and 17.5 ± 9.7 mIU/L in Q4 (*p* < 0.05). Overall, the cognitive and functional measures were not significantly different between the two groups. Q4 subjects showed lower levels of LDL and HDL cholesterol, as well as higher triglyceride levels compared with Q1 subjects. The percentage of subjects on statin treatment and antihypertensive treatment was similar between the two groups. Moreover, Q4 subjects had significantly higher BMI compared with Q1 subjects.

## Discussion

In this study, we demonstrated that, in a non-diabetic population of AD subjects, peripheral insulin resistance measured as HOMA2 is associated with reduced cerebral glucose metabolism measured by [18F]FDG PET and with reduced grey matter volume suggesting insulin resistance has significant influence on neurodegeneration even in non-diabetic AD patients. Interestingly, small vessel disease in our population was not affected by insulin resistance suggesting insulin resistance can exert its effect independent of cerebral small vessel disease in non-diabetic AD population.

This is the first study in the non-diabetic AD population to highlight the association of insulin resistance with hypometabolism and grey matter atrophy, and insulin resistance in our cohort was not associated with white matter lesions. It has been suggested that peripheral insulin resistance might mirror central insulin resistance in diabetic patients; however, not many studies have evaluated the influence of insulin resistance in non-diabetic AD patients. It is proposed that insulin resistance might precede the development of AD in patients predisposed to diabetes [28, 29]. While, in diabetic patients, PET studies have demonstrated that greater insulin resistance is associated with an AD-like pattern of reduced cerebral glucose metabolic rate in frontal, parietotemporal and cingulate regions [30], our cohort did not include subjects with type 2 diabetes and thus the confounding factors related to diabetes, such as systemic diabetic complications and the effects of anti-diabetic medications, are not present in the results of our study. Indeed, other comorbidities of type 2 diabetes (hyperglycaemia, inflammation, dyslipidaemia, renal failure, hypertension) have their own complex effects on brain function. In this study of non-diabetic subjects, the conclusions we draw relate to peripheral insulin resistance, devoid of the interactions with the other diabetes comorbidities and white matter changes.

A study in a cohort of non-diabetic mild cognitive impairment (MCI) subjects has shown an association between HOMA2-reduced performance in verbal fluency tests [31], but the authors did not evaluate AD biomarkers. Willette et al. evaluated AD subjects from the Alzheimer’s Disease Neuroimaging Initiative (ADNI) cohort which included diabetic subjects and showed an inverse association between HOMA2 and brain metabolism [32]. To our knowledge, no other studies have evaluated the association of insulin resistance with AD biomarkers in non-diabetic patients. Thus, our results further support the hypothesis that the action of insulin in the brain goes beyond glycaemic control and diabetes-related changes in the brain. Furthermore, although insulin resistance increases with age and, in older adults without dementia cortical insulin concentration is decreased, in our population the association between insulin resistance brain hypometabolism and grey matter volume was independent of age and gender.

It is known that increased insulin signalling in the brain might reduce Aβ accumulation; insulin has also been reported to enhance Aβ clearance from the brain [33]. Moreover, insulin resistance is known to be associated with increased levels of proinflammatory cytokines such as C-reactive protein, tumour necrosis factor- (TNF-) α, interleukin- (IL) 1, and IL-6 [13]. IL-6 and C-reactive protein are linked to Aβ plaque deposition and progression, and it is known that patients under chronic nonsteroidal anti-inflammatory therapy have reduced incidence of AD. Another relevant aspect is represented by the proinflammatory role of astrocytes and microglia surrounding Aβ plaques that are responsible for irreversible neuronal damage if chronically activated. Interestingly, insulin seems to have anti-inflammatory effects directly suppressing proinflammatory cytokines and inducing anti-inflammatory mediators, as demonstrated in both preclinical and clinical studies [34]. Nasal application of insulin, which allows it to enter the brain more directly than other administration routes, has clear effects on attention and memory formation. Pilot trials of intranasal insulin in patients with AD and MCI improved cognition and functional ability compared with placebo, while cerebral [18F]FDG uptake significantly worsened in the placebo-treated group [15]. Recently, the long-lasting insulin, detemir, has been tested for intranasal administration in AD and MCI, showing a treatment effect for the memory composite outcome compared with placebo. Like previous studies, this effect was moderated by the APOE status [35]. However, evidence from a recent trial of intranasal insulin in AD subjects did not show benefit in terms of cognitive outcomes at 12 months [18].

In our cohort, a higher insulin resistance index was associated with lower hippocampal rCMRGlc, but not with hippocampal atrophy or cerebrovascular (small vessel) disease, and one could argue that the mechanisms underlying hippocampal atrophy or white matter changes in our non-diabetic population are independent of peripheral insulin resistance. In particular, the available evidence suggests that hippocampal atrophy is a hallmark and probably the starting point of the pathogenesis of AD. Deposition of tau protein, formation of neurofibrillary tangles and accumulation of amyloid contributes to hippocampal atrophy, together with damage caused by several other factors. In our study, insulin resistance was negatively associated with total grey matter volume. It is known that diabetic patients show increased brain atrophy compared with healthy controls, but the underlying mechanisms are not well explained and might go beyond insulin resistance. Type 2 diabetes has been independently associated with reduced frontal and parietal cortical thickness in the ADNI cohort [36] and a voxel-based morphology study has demonstrated that the grey matter volume loss attributable to type 2 diabetes involves mainly temporal lobe, parahippocampus, cingulate gyrus and medial frontal regions [37]. Indeed, studies in patients with type 2 diabetes have not shown increased AD-like biomarkers in their CSF [38] and no difference in CSF biomarkers has been demonstrated between insulin resistance and non-insulin resistance in cognitively healthy individuals [39]. The cerebrovascular burden in our cohort that we measured from WML volume was not associated with insulin resistance. An association with age was found, as expected, and interestingly an independent positive relationship with serum triglyceride levels, as also shown in other elderly populations [40]. Previous studies have shown variable associations between insulin resistance and AD-like brain changes. In a non-diabetic, cognitively intact cohort of post-menopausal women at risk for AD, a significant negative relationship between HOMA-IR and right and total hippocampal volume, overall cognitive performance and selective tests of verbal and non-verbal memory was demonstrated [41]. A voxel-based morphometry study in middle-aged cognitively normal adults showed that higher insulin resistance predicted less grey matter at baseline and 4-year follow-up in medial temporal lobe, prefrontal cortices, precuneus and parietal gyri [42]. Others have reported that, in early AD, higher insulin response to intravenous glucose tolerance test was associated with less longitudinal decline in cognitive performance, and less regional atrophy in the bilateral hippocampi and cingulate cortices [43]. Interestingly, a recent study on plasma exosomal biomarkers of insulin resistance found a negative association between a marker of insulin resistance and bilateral parietal-occipital junction and right middle temporal gyrus volumes in patients with early AD [44].

In asymptomatic APOE e4 carriers, insulin resistance has been associated with higher CSF levels of tau, suggesting that insulin resistance might contribute to neurodegeneration [45]. In subjects with family history of AD, higher insulin resistance has been associated with reduced cerebral glucose metabolism [46], as well as changes in CSF Aβ and tau and worse memory performance, especially in APOE e4 carriers [47]. Recently, it has been shown that insulin sensitivity, together with adherence to a Mediterranean-style diet, were positively associated with MRI-based cortical thickness in middle-aged adults [48]. In addition to these observations, WMLs, detected as a high-intensity area in FLAIR MRI, reflect small vessel damage in periventricular and subcortical areas of the brain. They are associated with progression of cognitive decline and studies in diabetes have shown that WML severity is correlated with cognitive decline, suggesting that insulin resistance might be the exacerbating factor [49]. The findings from our study suggest that factors related to diabetes other than insulin resistance might contribute to cerebrovascular pathology.

The age range for our study population is 55–85 years, including a proportion of subjects that could be classified as early-onset Alzheimer’s disease (EOAD). We did not see a difference between the insulin resistance in this group compared with the older age group. Some studies in subjects with nmEOAD without AD family history have shown more atrophy and more prominent cortical hypometabolism compared to LOAD [50]. Moreover, age is known to affect IR. Indeed, in older persons, age-related changes in body composition, mitochondrial dysfunction, increased inflammation, oxidative stress and modified activity of insulin sensitivity regulatory enzymes increase the risk of developing IR [51]. In our study population, however, age did not seem to affect the relationship between peripheral IR and hippocampal glucose metabolism. Moreover, there was no significant difference in age between subjects with low or high HOMA2 index.

Kim et al. demonstrated an inverse association between insulin resistance and performance on verbal fluency tests in non-diabetic subjects with MCI, with a significant interaction of the APOE e4 carrier status [31]. Data from diabetic patients suggest that higher insulin resistance is associated with lower cognitive performance [52], while recent longitudinal evidence suggests that insulin resistance is an independent predictor of poor verbal fluency for 11 years follow-up [53]. Although the results of our study are not conclusive regarding the role of insulin resistance in non-diabetic AD, our findings highlight the fact that insulin resistance might be a modifiable risk factor and could be a potential therapeutic target in dementia prevention, also in non-diabetic subjects. However, it should also be noted that the relationship between AD pathology and insulin resistance could be bidirectional: the protracted accumulation of Aβ and tau with consequent neurodegeneration in areas of the brain such as the hypothalamus in AD could alter the central regulation of body energy metabolism and promote systemic insulin resistance [54].

In our cohort, subjects in the highest quartile of HOMA2 presented with higher BMI, higher serum triglyceride levels and lower HDL cholesterol levels. The proportion of subjects on antihypertensive treatment was not significantly different between subjects with low or high HOMA2 index. IR measured by HOMA2 represents a component and an underlying factor of the metabolic syndrome, together with hypertension, obesity and dyslipidaemia. These risk factors cluster together and are linked to increased risk of diabetes and coronary heart disease [55]. Studies have shown that fasting hyperinsulinemia/insulin resistance preceded the development of other aspects of the metabolic syndrome including hypertension, hypertriglyceridemia and reduced HDL. Moreover, even after adjusting for obesity, significant relationships between insulin and the other factors have been observed [56]. Therefore, it would be difficult to evaluate the modifying effects of single metabolic factors on brain glucose metabolism, as they are all part of the same cluster of metabolic alterations and are correlated to each other.

### Limitations

One of the limitations of our study is the unknown Aβ or tau status of our subjects, thus a global amyloid/tau/neurodegeneration (A/T/N) classification, as suggested by the most recent guidelines, was not possible [57]. Moreover, the data on APOE genotype are not available in this cohort. HOMA2 provides a measure of insulin resistance derived from fasting blood glucose and insulin levels, as well as measures of β-cell function and insulin sensitivity, which employs a non-linear model and constitutes the updated and recommended version of the original HOMA model [58]. However, the euglycemic-hyperinsulinemic clamp would provide a closer measure of insulin sensitivity, but was beyond the scope of this study. Although majority of the scans were performed around mid-day following 4 h fasting in a relatively short time window during the day (all the scans were performed between 10 AM and 3 PM), we cannot exclude that the actual time of the day might have some influence on glucose and insulin levels, which are known to follow a diurnal pattern [59]. However, variations in secretion are most evident when comparing early morning versus evening measurements [60, 61]. Lastly, the cross-sectional nature of this work does not allow for evaluating the predictive role of insulin resistance on AD-related biomarker change. Our findings provide further evidence on the association between peripheral insulin resistance and brain glucose metabolism and atrophy in AD, even in non-diabetic subjects. In addition to that, the disappointing results from most anti-amyloid therapeutic strategies in AD clearly indicate that other pathways need to be explored in the search for disease-modifying agents in AD. The pathophysiological connections between AD and type 2 diabetes provide further evidence for novel therapeutic strategies.

## Conclusion

In this non-diabetic AD population, insulin resistance was negatively associated with hippocampal glucose metabolism, along with total grey matter volume. This highlights the influence of peripheral insulin resistance on the neurodegenerative process even in non-diabetic AD subjects. The findings from our study support the importance of metabolic risk factors in non-diabetic AD patients and highlight the potential role of novel therapeutic strategies targeting insulin resistance.

## Supplementary Information

**Additional file 1: **Supplemental Figure 1. Correlation between WMLs and serum triglyceride levels. WMLs volume (in mL) was positively correlated with serum triglyceride levels (in mmol/L) (R2 = 0.22, *p* = 0.00).

**Additional file 2:.** Table S1. Inclusion and exclusion criteria.

## Data Availability

The datasets used and analysed during the current study are available from the corresponding author on reasonable request.
