# Supplementary figures and images for: Does insulin resistance influence neurodegeneration in non-diabetic Alzheimer’s subjects?
Source: Alzheimers Res Ther. 2021 Feb 17;13:47. doi: 10.1186/s13195-021-00784-w (PMC7890851; doi:10.1186/s13195-021-00784-w)

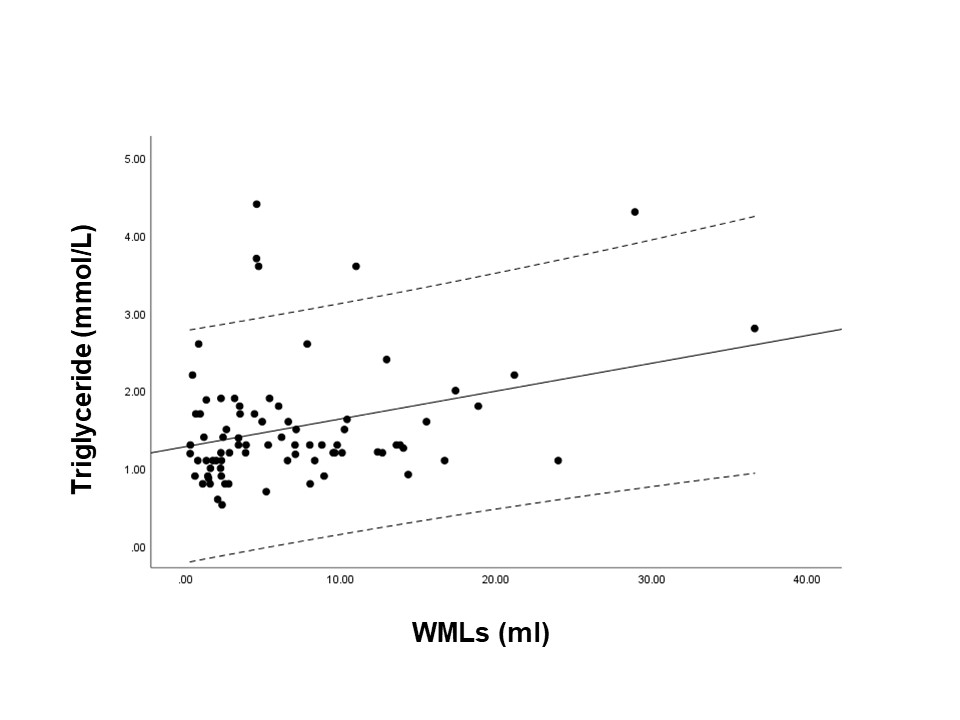

Supplement: Supplementary file 1 — Additional file 1: Supplemental Figure 1. Correlation between WMLs and serum triglyceride levels. WMLs volume (in mL) was positively correlated with serum triglyceride levels (in mmol/L) (R2 = 0.22, p = 0.00). [file 13195_2021_784_MOESM1_ESM.jpg]
