# Supplementary material for: Does insulin resistance influence neurodegeneration in non-diabetic Alzheimer’s subjects?
Source: Alzheimers Res Ther. 2021 Feb 17;13:47. doi: 10.1186/s13195-021-00784-w (PMC7890851; doi:10.1186/s13195-021-00784-w)
Supplement: Supplementary file 2 — Additional file 2:. Table S1. Inclusion and exclusion criteria. [file 13195_2021_784_MOESM2_ESM.docx]

**Table 1. Inclusion and exclusion criteria**

| **Inclusion criteria** |
| --- |
| 1. Capable of giving and capacity to give informed consent |
| 2. An individual who can act as a reliable study partner with regular contact (a combination of face-to-face visits and telephone contact is acceptable) who has sufficient interaction with the participant to provide meaningful input into rating scales and, if necessary, supervise or perform the injections, as judged by the investigator |
| 3. Diagnosis of probable AD disease according to National Institute on Aging–Alzheimer’s Association (NIA-AA) criteria or National Institute of Neurological and Communicative Disorders and Stroke–Alzheimer’s Disease and Related Disorders Association (NINCDS-ADRDA) criteria |
| 4. Age from 50 years |
| 5. Mini-Mental State Examination score of ≥ 15 and CDR-Global score of 0.5, 1, or 2 with capacity to consent, and the clinician anticipates that the participant will have capacity to complete the study |
| 6. Rosen Modified Hachinski Ischemic score ≤ 4 |
| 7. On stable medication for 2 months before the screening visit; on or off cholinesterase inhibitors |
| 8. Fluency in English and evidence of adequate premorbid intellectual functioning |
| 9. Likely to be able to participate in all scheduled evaluations and complete all required tests |
| **Exclusion criteria** |
| 1. Patients on treatment for diabetes mellitus |
| 2. Any contraindications to the use of liraglutide as per the summary of product characteristics (hepatic impairment, renal impairment with chronic kidney disease stage 4 and above (eGFR < 30 ml/min/1.73 m^2^), or inflammatory bowel disease). Patients with eGFR < 45 ml/min/1.73 m^2^ will have their renal function monitored very closely |
| 3. Significant neurological disease other than AD that may affect cognition |
| 4. MRI/CT showing unambiguous aetiological evidence of cerebrovascular disease with regard to their dementia or vascular dementia, fulfilling National Institute of Neurological Disorders and Stroke -Association Internationale pour la Recherche et l'Enseignement en Neurosciences (NINCDS-AIREN) criteria |
| 5. Current presence of a clinically significant major psychiatric disorder (e.g., major depressive disorder) according to the criteria of the *Diagnostic and Statistical Manual of Mental Disorders*, Fourth Edition (DSM-IV) |
| 6. Current clinically significant systemic illness that is likely to result in the patient’s condition deteriorating or affect the patient’s safety during the study |
| 7. History of epilepsy, where seizures or treatment could have contributed to cognitive impairment |
| 8. Treatment with immunosuppressive medications (e.g., systemic corticosteroids) within the last 90 days (topical and nasal corticosteroids and inhaled corticosteroids for asthma are permitted) or chemotherapeutic agents for malignancy within the last 3 years |
| 9. Myocardial infarction within the last 1 year |
| 10. History of cancer within the last 5 years, except localised skin cancer |
| 11. Other clinically significant abnormalities on physical, neurological, or laboratory examination that could compromise the study or be detrimental to the patient |
| 12. History of alcohol or drug dependence or abuse within the last 2 years |
| 13. Current use of anticonvulsant, anti-Parkinson’s disease medication |
| 14. Use of experimental medications for AD or any other investigational medication or device within 60 days. Participants who have been involved in a monoclonal antibody study are excluded unless it is known that they were receiving placebo in that trial |
| 15. Women of childbearing potential (women who could become pregnant will be required to use adequate contraception throughout the trial) |
| 16. Patients with a personal or family history of medullary thyroid carcinoma and patients with multiple endocrine neoplasia type 2 |
| 17. Any contraindications to MRI scanning |
